# Integrated Care Models for Older Adults with Depression and Physical Comorbidity: A Scoping Review

**DOI:** 10.5334/ijic.7576

**Published:** 2024-01-10

**Authors:** Laura Tops, Simon Gabriël Beerten, Mathieu Vandenbulcke, Mieke Vermandere, Mieke Deschodt

**Affiliations:** 1 Academic Centre of General Practice, KU Leuven, Kapucijnenvoer 7, Box 7001, 3000, Leuven, Belgium; 2 Department of Public Health and Primary Care, KU Leuven, Leuven, Belgium; 3 Department of Neurosciences, Leuven Brain Institute, KU Leuven, Leuven, Belgium; 4 Department of Geriatric Psychiatry, University Psychiatric Centre, KU Leuven, Leuven, Belgium; 5 Competence Center for Nursing, University Hospitals Leuven, Belgium; 6 Gerontology and Geriatrics, University Hospitals Leuven, Belgium

**Keywords:** collaborative care, integrated care, psychosomatic medicine, geriatric psychiatry, care models

## Abstract

**Objective:**

Multimorbidity is a growing challenge in the care for older people with mental illness. To address both physical and mental illnesses, integrated care management is required. The purpose of this scoping review is to identify core components of integrated care models for older adults with depression and physical comorbidity, and map reported outcomes and implementation strategies.

**Methods:**

PubMed, EMBASE, CINAHL and Cochrane Library were searched independently by two reviewers for studies concerning integrated care interventions for older adults with depression and physical comorbidity. We used the SELFIE framework to map core components of integrated care models. Clinical and organisational outcomes were mapped.

**Results:**

Thirty-eight studies describing thirteen care models were included. In all care models, a multidisciplinary team was involved. The following core components were mainly described: continuity, person-centredness, tailored holistic assessment, pro-activeness, treatment interaction, individualized care planning, and coordination tailored to complexity of care needs. Twenty-seven different outcomes were evaluated, with more attention given to clinical than to organisational outcomes.

**Conclusion:**

The core components that comprise integrated care models are diverse. Future studies should focus more on implementation aspects of the intervention and describe financial parts, e.g., the cost of the intervention for the healthcare user, more transparently.

## Introduction

Depression is the most common mental disorder in adults aged 60 and over, affecting approximately 13% of the world’s older population [1,2]. Depression is characterized by depressed mood, loss of motivation, lack of physical energy, inability to feel pleasure, sleep disturbance, decreased focus, and feelings of hopelessness. Depression accounts for 5, 7% of Years Lived with Disability [YLDs] among those over 60 years old, and is both underdiagnosed and undertreated in primary care settings [2]. People with mental illness have higher rates of hospitalization and emergency department use than patients with chronic physical conditions alone [3,4].

More than two-thirds of older patients attending psychiatric services have at least one physical illness, and more than half of those have at least two [5]. Multimorbidity is defined as the coexistence of multiple chronic conditions in one person at the same time, where one is not a known complication of the other. This is a growing challenge in older people with mental illness. The risk of developing cardiac disease, hypertension, stroke, diabetes, metabolic syndrome or obesity is around 40% higher for patients with depression than in the general population, and the risk of developing Parkinson’s disease is even doubled compared with people without depression [6,7,8,9,10,11,12].

Many barriers compromise the care for depressed older people with physical multimorbidity. Examples include the absence of care coordination, an incomplete medical file, the patient and family not being involved as partners, the constrained collaboration between care actors, stigma and prejudice toward the psychiatric patient, shortage of time and staff to organise somatic health care, lack of implementation of evidence-based guidelines and underuse of prevention and health promotion [6,7]. Effective management of multimorbidity requires integrated care, so that common risk factors and the bidirectional interaction between physical and mental illnesses, and the treatment for each, can be addressed together [11,13,14]. Integrated care is defined as structured efforts to provide coordinated, pro-active, person-centred, multidisciplinary care by two or more well-communicating and collaborating care providers either within or across sectors. To realize such integrated care, a paradigm shift from disease- to person-centredness is necessary in service delivery, management, and funding [15]. The integration of mental and physical health care is nowadays top priority in national and international policy documents [3,6,7,16,17,18]. Despite the growing body of literature for integrated care models in chronic care, evidence on the effectiveness of integrated care for multimorbidity is still limited [19,20,21,22].

The WHO recently issued an Integrated Care for Older People [ICOPE] approach to guide healthcare systems and services in better supporting the capacities of older people. They described key elements of integrated care models for older people: comprehensive assessments and integrated care plans; shared decision-making and goal setting; support for self-management; multidisciplinary teams; unified information or data-sharing systems; community linkages or integration and supportive leadership, governance, and financing mechanisms [23]. A recent review found that key elements of existing integrated care models for older adults comprise some elements of the ICOPE approach, namely: multidisciplinary team care, comprehensive assessment, and case management [24]. However, although core components of integrated care models have been suggested by various scholars and institutions in the healthcare domain, there are no clear guidelines on how to incorporate them into a real-life healthcare setting [24].

A recent meta-analysis on nurse-led integrated care models found that implementation strategies are often under-reported [25]. To adopt and maintain a complex intervention in a real-world setting, attention should be paid to implementation strategies such as theories, models, or frameworks [26,27]. The success of an intervention is reliant on the level of implementation in a specific context [28].

Unlike Struckmann et al. [29], who focused on the general adult population without specific emphasis on “depression,” our review solely focuses on integrated care models in in older adults dealing with both depression and physical multimorbidity, regardless of care setting. Leithaus et al. [30] focused on transitional care between hospital and home models, while our scope extends to a wider array of integrated care approaches. In comparison to Prajankett & Markaki [31], whose focus was limited to the US and Thailand, and excluded secondary and tertiary care settings, our study offers a more comprehensive global perspective. Additionally, our analysis includes generalist nurses, nurse midwives, and physician assistants, contributing to a more nuanced understanding of the subject matter.

The primary aim of this scoping review is to identify the core components of integrated care models applied to older patients with major depression and physical comorbidity. Secondarily, we will evaluate the effectiveness of these models on the clinical and organisational outcomes and map the reported implementation strategies.

## Methodology

We followed the methodological guidance for scoping reviews of Arksey and O’Malley’s [32] and used the PRISMA-SCR [Preferred Reporting Items for Systematic reviews and Meta-Analyses extension for Scoping Reviews] checklist for reporting the review process [27]. The protocol of this scoping review has been published prior to the start of the screening process [33].

### Search strategy

We searched PubMed, EMBASE, CINAHL and Cochrane Library for papers published from inception of the database until the 18^th^ of May 2022. A comprehensive search string was developed in PubMed assisted by a research librarian using MeSH terms and free text words related to “older patients” AND “depression” AND “integrated care” [See Annex 1]. We then adapted the search string for the other databases. Backward and forward reference searching of the included articles was performed to identify other relevant articles, e.g., study protocol papers. One researcher explored the grey literature by conducting a search via ClinicalTrials.gov database and the International Clinical Trials Registry Platform.

### Eligibility criteria

In accordance with the scoping review approach [34], we developed the in- and exclusion criteria for the scoping review iteratively. The final set of criteria are reported here. We included studies published in English or in Dutch targeting people aged 60 years or older with a major depression diagnosis according to a validated scale and having at least two additional physical comorbid conditions. These physical comorbidities had to meet the definition of a non-communicable disease [NCD] according to the World Health Organisation [WHO] [35]. We included all types of original quantitative and qualitative study designs concerning integrated care interventions [including study protocols]. Studies describing single-component interventions including solely an online or telephone-based intervention component were excluded. Studies including patients with a neurocognitive disorder [e.g., dementia] were excluded.

### Study selection

We first imported the results of the systematic search in all databases in an Endnote file to delete all duplicate references. We then used Zotero as a reference manager to support the independent selection process of the two reviewers [L.T. & S.G.B.]. All titles and abstracts were screened and subsequently a full-text screening of the potentially relevant papers was conducted. When there was disagreement regarding inclusion, a third researcher [M.D.] was consulted to discuss and decide upon final inclusion in the scoping review. No additional references were added by searching the grey literature.

### Data extraction and synthesis

We used the Joanna Briggs Institute [JBI] Reviewer’s manual 2020 as a guide to chart the data [36]. Two researchers [L.T. & S.G.B.] extracted the following data from the included articles: the authors and year of publication, the name of the care model, the country where the study was conducted, the study design, the inclusion criteria, and the sample [See Annex II]. We also extracted data regarding the core components of the care model. The international SELFIE consortium developed a conceptual framework that structures relevant concepts in integrated care for multimorbidity and can be applied by different stakeholders to guide development, implementation, description, and evaluation [37]. We used the domains related to the micro-level of the Sustainable intEgrated chronic care modeLs for multi-morbidity: delivery, FInancing, and performancE [SELFIE] framework. These domains are service delivery, leadership & governance, workforce, financing, technology and information & research (Table 1). We also listed all the clinical and organizational outcomes that were measured in the included studies and marked whether significant effects had been reported (Table 2). Lastly, we extracted data related to the implementation of the care models. This includes the reporting of contextual analyses, stakeholder involvement, the use of a logic model, the implementation strategies, the conduct of process evaluations or the use of a framework to guide the implementation pathway [26,28,38]. Logic models can be of great help to implement a complex intervention successfully, as they represent the causal processes of interventions. They describe the resources, activities, outputs, and outcomes of an intervention [39].

**Table 1:** Core components of the integrated care models.


CARE MODEL	SERVICE DELIVERY							LEADERSHIP & GOVERNANCE			WORKFORCE			FINANCING		TECHNOLOGY		INFORMATION & RESEARCH		
					
	PERSON-CENTRED	TAILORED HOLISTIC ASSESSMENT	SELF-MANAGEMENT SUPPORT	PRO-ACTIVE	INFORMAL CAREGIVER INVOLVEMENT	TREATMENT INTERACTION	CONTINUITY	SHARED DECISION-MAKING	INDIVIDUALIZED CARE PLANNING	COORDINATION TAILORED TO COMPLEXITY OF CARE NEEDS	MULTIDISCIPLINARY TEAM	NAMED COORDINATOR	CORE GROUP	OUT OF POCKET COSTS	FINANCIAL INCENTIVES	ELECTRONICAL MEDICAL RECORDS & PATIENT PORTALS	E-HEALTH TOOLS OR TELEMEDICINE	INDIVIDUAL RISK PREDICTION	EFFECTIVE USE OF INDIVIDUAL LEVEL DATA, I.E., AUTOMATIC NOTIFICATION OF ED VISIT	COMPUTERIZED ALGORITHMS THAT RECOMMEND CARE PATHWAYS

BRIGHTEN [50,53]	X	X	X	X		X	X	X	X	X	X	DCM	DCM, PCP, GPsychi, C, Nut, O, Phys, SW, Gpsycho			X	X			

CAREPATH [42,54]	X	X	X	X	X	X	X	X	X	X	X	DCM	DCM, PCP			X	X			

CASPER plus [43,55,56]	X	X	X	X	X	X	X	X	X	X	X	CM	CM, PCP, MHS		X	X	X	X	X	

CEPIS [51]	X	X	X	X		X	X	X	X	X	X	CM	CM [N], GP, MHS			X				

DCM China [41,57]	X	X	X	X	X	X	X		X	X		X	CM	CM, PCP, MHS, N			X			

GermanIMPACT [45,58]	X	X	X	X		X	X	X	X	X	X	CM	CM, PCP, MHS, P			X				

IMPACT [49,59,60,61,62,63,64,65,66,67,68,69,70,71]	X	X	X	X		X	X	X	X	X	X	DCM	DCM, PCP, Psychi, P	X	X	X	X			

IMPACT-DP [47]	X	X	X	X		X	X	X	X	X	X	CM	CM [N], PCP, GPsychi			X	X			

Improving Depression Treatment for Older Minority Adults in Public Sector Care [46]	X	X	X	X		X	X	X	X	X	X		PCP, Psych, SW	X						

Multifaceted shared care intervention for late life depression in residential care [52]	X	X	X	X		X	X	X	X	X	X	PCP	PCP, Residential staff, N, MHS							

PRIDE [48]	X	X	X	X		X	X	X	X	X	X	CM	CM [N], PCP, GPsychi			X	X			

PROSPECT [40,72,73,74,75,76,77]	X	X		X		X	X	X	X	X	X	DCM	DCM, PCP, GPsychi				X	X		

The Seniors Outpatient Community-Based Collaborative Care Model [44]	X	X	X	X	X	X	X		X	X	X	CM	CM, PCP, GPsychi, G,				X			


**GPsychi:** geriatric psychiatrist; **GPsycho:** geriatric psychologist; **PCP:** primary care physician; **N:** nurse; **MHS:** mental health specialist; **Psych:** psychotherapist; **Psychi:** psychiatrist; **SW:** social worker; **P:** patient; **G:** geriatrician; **[D]CM:** [depression] care manager; **C:** chaplain; **Nut:** nutritionist; **O:** occupational therapist; **Phys:** physical therapist.

**Table 2:** Reported clinical and organisational outcomes.


	CLINICAL OUTCOMES																						ORGANISATIONAL OUTCOMES					N
		
	DEPRESSION SYMPTOMS	SEVERITY OF DEPRESSION/REMISSION	DEPRESSION RESPONSE TO TREATMENT	ANXIETY	SUICIDAL IDEATION	FUNCTIONAL STATUS	MENTAL HEALTH	ADLS	IADLS	SF-12	STIGMA	PATIENT SATISFACTION	HEALTH-RELATED QUALITY OF LIFE	PAIN	ANALGETIC USE	ANTIDEPRESSANT MEDICATION USE	HOPELESSNESS	SELF-MANAGEMENT	RESILIENCE	ACTIVITY SCHEDULING	ENGAGEMENT IN ACTIVITIES	VITAL STATUS	THERAPY ATTENDANCE	ED VISITS	GP VISITS	USE OF MENTAL HEALTH SERV	HEALTHCARE COSTS	27

Alexopoulos 2005		**+**		0	0	**+**	0									0	**+**											7

Alexopoulos 2009		**+**	**+**		**+**																							3

Bogner 2005		0	0																									2

Bogner 2007		0			0			0														0						4

Bogner 2012		**+**			0																							2

Bosanquet 2017		**+**		**+**		0	0						0			0			**+**								0	8

Bruce 2004		**+**	**+**		**+**																							3

Bruce 2015		**+**				0		0	0																			4

Callahan 2005						**+**			**+**	**+**																		3

Chen 2015	**+**	**+**	**+**							**+**	**+**	**+**																6

Chew-Graham 2007	**+**	0				0								0														4

Emery 2012	**+**					0	**+**																					3

Fann 2009	**+**	**+**	**+**			**+**						**+**	**+**			**+**										**+**		8

Harpole 2005		0	0	0			0						0			0												6

Hunkeler 2006	**+**	**+**	**+**			0	**+**			0		**+**	**+**			**+**		**+**								0		11

Khambaty 2018		**+**														0												2

Lin 2006		0				**+**								**+**	0					0								5

Lin 2003		**+**				**+**							**+**	**+**														4

Llewelyn Jones 1999	**+**					0			0									0						0	0			6

Ng 2020		**+**	**+**				0					**+**	**+**															5

Penkunas 2015		**+**																					**+**					2

Riebe 2012		**+**																		**+**	**+**							3

Schulberg 2007		0			0																							2

Shulman 2021		**+**		**+**		0																						3

Thielke 2007		0												0														2

Unutzer 2002	**+**	**+**	**+**			**+**						**+**				0											0	7

Unutzer 2008		**+**				**+**						**+**		**+**				**+**		**+**								6

Van LeeuwenWilliams 2009	0	0	0	0	0	0	0							0												0		9

Williams 2004		**+**				**+**										0		0										4

**N**	8	26	10	5	7	16	7	2	3	3	1	6	6	6	1	8	1	4	1	3	1	1	1	1	1	3	2	


0 = non-significant; **+** = significant.

## Results

### Search results

**Figure 1:**
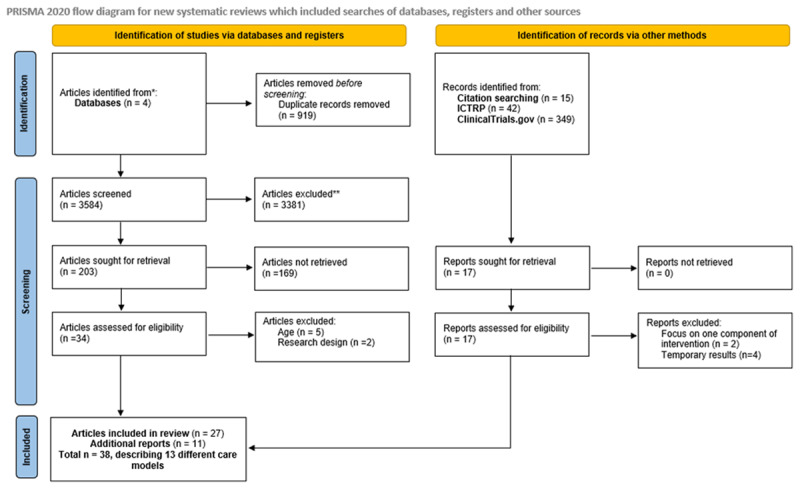
Adapted PRISMA Flow Diagram.

We identified 4503 articles through database searching. After removing duplicates, we included 123 articles through title and abstract screening. Eventually, 27 articles met all inclusion criteria. We identified 11 additional articles via snowball inclusion (Figure 1). In total, we identified 38 articles based on 13 care models.

### Characteristics of included studies

The study designs were: randomized controlled trials [n = 21], observational studies [n = 6], qualitative studies [n = 3], study protocols [n = 3], and feasibility studies [n = 5]. Thirteen care models were evaluated in North America [n = 7], Europe [n = 3], Asia [n = 2] and Australia [n = 1]. The sample size ranged from 11 to 4633 participants [See Annex: II].

### Core components of the care models

#### Service delivery

All 13 care models applied a person-centered approach by performing a tailored holistic assessment at the beginning of the intervention. All but one care model [40] stimulated patients to take control over their care planning by educating them on self-management strategies. A pro-active approach to the care planning was used in every care model. Informal caregivers were engaged in four out of thirteen care models [41,42,43,44]. The interaction of treatment [e.g., polypharmacy, guideline interaction] was assessed in every care model. Continuity of care was ensured by follow-up of the intervention in all care models through administrating telephone calls [n = 2] [44,45], in-person assessments [n = 2] [40,46], or using both techniques [n = 3] [43,47,48]. In six care models, the type of follow-up was not well described (Table 1).

#### Leadership & governance

In eleven of the thirteen care models, shared decision-making was described as a core component of the intervention [40,42,43,45,46,47,48,49,50,51,52]. The care manager was responsible for managing the individualized care planning of the patient [n = 13] and tailoring the coordination to the patient’s care needs whenever necessary [n = 13].

#### Workforce

A multidisciplinary team was involved in all care models and all but one [46] had a dedicated care manager. The combination of team members varied per care model. In five care models, the core group consisted of a care manager, a primary care physician and a psychiatrist or mental health specialist [40,43,47,48,51]. The patient was explicitly part of the core group in two care models [45,49]. A geriatrician, psychotherapist, nutritionist, chaplain, geriatric psychologist, occupational therapist, and physical therapist were involved in one care model, respectively [50].

#### Financing

Six care models reported explicitly on the reimbursement of the intervention costs for participants, all intervention related costs were covered by the project itself [40,41,42,43,50,51]. Regarding the financial incentives to motivate participants to take part in the integrated care intervention, two care models reported on providing a financial compensation for participants [43,49]. One care model highlighted that their intervention was partly covered by the project. More specifically, visits with care managers and team psychiatrists were free of charge, however patients were responsible for all other health care costs e.g., antidepressant medications [49].

#### Technology

All but two [46,52] care models employed some variant of technology, as defined by the SELFIE framework [37], as part of their interventions. Telephone calls were used to follow patients’ progress throughout the intervention [n = 8] [40,41,43,44,45,47,48,49]. A few care models also used a web-based information system [n = 3] [40,43,70].

#### Information & research

Two out of thirteen care models reported on paying attention to individual risk prediction [40,43]. One care model reported on a computerized case management system called PC-MIS which facilitates symptom monitoring [43]. None of the care models reported on using computerized algorithms that recommend care pathways.

### Outcomes and effectiveness of the studies

A total of 27 outcomes were measured in all studies evaluating the care models. Outcomes were divided in clinical outcomes [n = 22] and organisational outcomes [n = 5]. The number of reported outcomes in the quantitative studies varied between two and ten per study (Table 2). Depression outcomes [n = 44] were by far the most reported outcome in all studies: severity of depression/remission [n = 26], response to treatment [n = 10], and depressive symptoms [n = 8]. Then followed by functional status [n = 16], antidepressant use [n = 8] and mental functioning [n = 7]. In contrast, organisational outcomes were less often reported. Therapy attendance, emergency department visits and GP visits were all measured in only one study.

Significant differences were observed for depression, functional status, mental functioning, stigma, patient satisfaction and health-related quality of life in patients subject to the Depression Care Management [DCM] intervention [41]. In the PRIDE care model [48], a significant beneficial impact was found on depression symptoms. Besides depression symptoms, BRIGHTEN [50] found significant results for mental functioning. Severity of depression, anxiety, and resilience were significant in the CASPER plus intervention [43]. The Seniors Outpatient Community-Based Collaborative Care Model [44] was effective on severity of depression and anxiety. In addition to severity of depression, IMPACT-DP [47] found significant effects on functional status, patient satisfaction, self-management, pain and activity scheduling. CEPIS [51] was effective on the outcomes of depression symptoms, depression severity, patient satisfaction and health-related quality of life. Depression severity was significant for the CAREPATH [42] intervention. The PROSPECT intervention found significant outcomes for depression severity [40,74,77,78], response to treatment [40,74], suicidal ideation [40,74], functional status [78], and hopelessness [78]. The IMPACT trial found significant results for: depression symptoms, depression severity, response to treatment, functional status, mental functioning, Instrumental Activities of Daily Living [IADL], patient satisfaction, health-related quality of life, pain, self-management, activity scheduling, engagement in activities and therapy attendance [49,59,61,62,64,65,66,68,71].

#### Implementation outcomes

Ten out of thirteen care models reported on implementation outcomes [41,43,44,45,46,47,48,49,50,51]. Three care models explicitly stated that they used the IMPACT intervention [49] as a basis for developing their own care model. GermanIMPACT [45] and IMPACT-DP [47] adapted the IMPACT intervention which originated from the United States and tailored the intervention to their own specific context. Furthermore, BRIGHTEN [79] based their intervention also on PROSPECT and PRISM-E, yet none of the studies described if they conducted a contextual analysis to adapt the care model.

The CASPER plus study explicitly stated that the researchers were informed by users and carers of mental health services throughout the study. Older adults, together with their carer representative, were involved in a patient and public involvement group [43,55]. IMPACT-DP was co-designed by adapting the original IMPACT program and involving key stakeholders in the designing process of the intervention [47].

None of the studies reported on using a logic model. Seven out of thirteen care models conducted a process evaluation [41,44,46,47,48,49,51]. Five of those used a qualitative research approach [focus group discussions or interviews] [44,46,47,48,56,70]. Educating healthcare professionals about the collaborative care model was found crucial for effective implementation [56]. More specifically, educating patients [and their network] about the different aspects and involved actors of the care model [47], and elucidating the usage of self-help material was found to be of importance [48]. Another study pointed out that the accessibility of a psychiatrist could not always be guaranteed on a frequent basis. Barriers to this were, among others, time constraints, distances, and traffic [70]. Two studies reported on the limited timeframe of the intervention and would opt for a prolonged intervention [44,47]. Two studies performed a process evaluation through quantitative methods. Participants were asked how satisfied they were with the intervention [41,51]. One study used both qualitative and quantitative methods to assess process evaluation [49].

Only one study used an implementation framework as part of their evaluation. In the BRIGHTEN study the RE-AIM framework was used to evaluate the Reach, Efficacy, Adoption, Implementation and Maintenance of the intervention [50,80].

## Discussion

### Summary of results

In this scoping review, we included 38 studies, describing 13 different care models. A total of 27 outcomes were described in the interventions. Core components on service delivery, leadership & governance and workforce were well described by the different studies. Person-centeredness tailored holistic assessment, pro-activeness, treatment interaction, continuity, individualised care planning, coordination tailored to complexity and multidisciplinary team were mentioned in every care model, although not in detail. In contrast, elements on financing and information & research were poorly described. A review on nurse-led integrated care models found similar results concerning the description of the core components [25].

### Comparison with other literature

In all care models, a multidisciplinary team was present. The majority [n = 12] of the care models also described having a care manager. According to the definition of integrated care models by the World Health Organisation, a coordinated multidisciplinary team is an indispensable component [81]. To bridge the gap between physical and mental healthcare, healthcare professionals should be stimulated to work closely together [82,83]. Although the composition of the core team varied, a primary care physician and a healthcare provider with psychiatric expertise were present in every multidisciplinary care team. It is of importance that patients take an active role in the care process, therefore having too many health care providers involved in the core team can be a threshold for patients to participate [84].

Self-management skills are a key component in managing a chronic disease [85,86]. Moreover, self-management can be of aid in reducing the severity of symptoms for depression [87]. Collaborative care can be effective in empowering individuals to take up self-management [88,89]. Although all care models reported to stimulate self-care as part of their intervention, only two care models investigated the impact on self-management outcomes. Self-management significantly improved in the IMPACT model [49].

Only four care models involved informal caregivers. A recent review found the same limited engagement of family in collaborative care programs for adults with depression and anxiety [90]. However, international consensus exists regarding the advantages of engaging families in healthcare practices [91]. Furthermore, it is important for professionals to be receptive to the involvement of families in the care planning process. The attitudes of health care professionals toward families can either help or hinder informal caregiver involvement [92].

Most of the care models used a form of technological assistance, mostly telephone follow-up or electronical medical records. The literature on effectiveness of telemedicine is controversial [93,94]. Ambiguity still exists regarding the safety and quality conditions of telephone consultations in general [93]. However, Information and Communication Technology [ICT] infrastructure can assist in collecting, storing, and sharing data among different stakeholders within the collaborative care model [95,96]. Especially in rural settings, telephone follow-up can be effective to overcome geographical problems and time constraints, thereby ensuring continuity of care [97]. E-health tools in general can contribute to the empowerment of the patient and stimulate self-management [37].

Depression was measured on three different levels: depression symptoms, severity of depression/remission and response to treatment. Hence, it was difficult to compare depression outcomes. Recently, a harmonized minimum set of outcomes for measuring depression across multiple healthcare settings was published. The authors defined ten outcome measures: all-cause mortality, death from suicide, improvement in depressive symptoms: remission, improvement in depressive symptoms: response, worsening in depressive symptoms: recurrence, adverse events, suicidal ideation and behaviour, depression-specific quality of life, depression-related resource use and work productivity [98]. **In the future**, research should make use of this harmonized set to facilitate comparison between depression outcomes of integrated care models.

Most of the reported outcomes [n = 22] were clinical outcomes. Organisational outcomes were less reported. **Future research** should focus more on describing organisational outcomes in detail, especially in terms of implementing interventions. As stated earlier, to implement a complex intervention successfully, logic models can be of great help. They describe the resources, activities, outputs, and outcomes of an intervention [39]. Other advantages in using logic models are for example: providing an overview of what to measure during the intervention, detecting missing gaps in the theory, aligning key stakeholders, and listing the evidence-based strategies [99,100,101,102]. Yet, none of the care models in this review utilized a logic model. However, more attention was given to process evaluation. Most of the studies conducted interviews or focus group discussions to evaluate the intervention in accordance with care users and/or care providers, to improve and adapt the care model. Process evaluation is a crucial part in the development process of a complex intervention, such as an integrated care model [103]. Due to the limited scope of the process evaluations, it was not possible to identify which core components were more effective than the others. **Future research** should focus on the relative impact of the individual core components by evaluating them through dedicated proximal outcomes and including process evaluations. Also, limited attention was given by the care models to implementation strategies. Only one care model used an implementation framework to evaluate their intervention [50].

The IMPACT trial [49] was developed and successfully implemented in primary care settings throughout the USA. Multiple care models try to tailor the IMPACT intervention to their context. A context analysis ensures that the intervention, in this case the care model, can be adapted properly to the specific setting [104,105]. To achieve a match between the complex intervention and the context, systematic adaption is needed. For example, ADAPT is a framework that provides a step-by-step guidance to adapt an evidence-based intervention to a new context [106]. Another framework that can be used is the revised version of the Medical Research Council [MRC] framework. Using a pluralistic approach, the framework provides four perspectives to guide the adaptation of the intervention: efficacy, effectiveness, theory based and systems [27]. GermanIMPACT [45] and IMPACT-DP [47] used the IMPACT intervention as a premise and adapted the intervention to their specific context. However, the articles in our search did not describe the details of the context analysis that took place.

Following the scoping review methods guidance, we refrained from conducting intricate statistical analyses to quantify the impact of studies or ascertain factors influencing positive intervention outcomes. Nevertheless, this scoping review established the presence of ample literature for potential analysis, which future research could address in a systematic review.

### Strengths and limitations

A thorough search of the grey literature was conducted. To minimize bias in setting up a search string, we consulted a research librarian. We have specifically chosen for a scoping review because they offer a valuable starting point for exploring research areas, providing a structured and organized approach to mapping existing literature and identifying research priorities [36]. By conducting the scoping review, we also encountered some limitations. First, integrated care was one of the three key concepts of the search string, however a uniform definition of ‘integrated care’ does not exist. Therefore, some relevant articles were potentially not discovered during our search. Secondly, we only evaluated care models for older people with major depressive disorder. Forms of subthreshold or mild depression were not included. Additionally, patients had to have at least two physical comorbid conditions. By using these strict eligibility criteria, we potentially missed other care models. Third, we restricted our data extraction to the predefined SELFIE framework, which could potentially lead to the omission of valuable information. Fourth, In the process of conducting this scoping review, we encountered notable challenges in effectively delineating the role of care manager’s responsibilities across diverse care models. These intricacies prompted us to opt for a more cautious approach, leading us to abstain from presenting an exhaustive representation of the care manager profile within the confines of our study. Lastly, the wide range of outcomes, follow-up periods, and study types posed challenges for direct comparisons, making it difficult to achieve a cohesive synthesis of numerical data. Delving into specific numerical details might have introduced confusion, which goes against the aim of providing a balanced overview. However, it is acknowledged that future research could explore the numerical and qualitative effectiveness of integrated care models to better comprehend their impact. This underscores the ongoing need for comprehensive evaluations in the ever-evolving landscape of integrated care.

## Conclusion

This scoping review identified 38 articles describing 13 different care models on older adults with depression and physical multimorbidity. However, financial aspects of the intervention were reported to a limited extent. There was a larger focus on clinical outcomes as opposed to organizational outcomes. Due to the lack of quantitative measurements in the process evaluations of the intervention studies, we could not distinguish which of the core components are more effective than others. Also implementation theories and context analysis were poorly described.

## Additional File

The additional file for this article can be found as follows:

10.5334/ijic.7576.s1 Annex. Annex I and Annex II.
