# Supplementary material for: Integrated Care Models for Older Adults with Depression and Physical Comorbidity: A Scoping Review
Source: Int J Integr Care. 2024 Jan 10;24(1):1. doi: 10.5334/ijic.7576 (PMC10786096; doi:10.5334/ijic.7576)
Supplement: Annex. — Annex I and Annex II. [file ijic-24-1-7576-s1.pdf]

Annex

Annex I: Search string Pubmed

**Aged**	"Aged"[Mesh] OR "Elder*"[tiab] OR "older patient*"[tiab] OR "older person*"[tiab] OR "older adult*"[tiab] OR "older subject*"[tiab] OR "aged person*"[tiab] OR "aged patient*"[tiab] OR "aged subject*"[tiab] OR "senior*"[tiab] OR "senium"[tiab] OR "old age"[tiab] OR "Oldest Old"[tiab] OR "Centenarian"[tiab] OR "Nonagenarian"[tiab] OR "Octogenarian"[tiab] OR "advanced age"[tiab] OR "geriatric*"[tiab] OR "geronto*"[tiab] OR “aged people”[tiab] OR “elder care”[tiab] OR “elderly subject”[tiab]
**Depression**	"Involutional Melanchol*"[tiab] OR "Involutional Psychos*"[tiab] OR "depressive syndrome*"[tiab] OR "depressive disorder*"[tiab] OR “depressive disorder”[Mesh]
**Integrated care**	"Delivery of Health Care, Integrated"[Mesh:NoExp] OR "Comprehensive Health Care"[Mesh] OR "Patient-Centered Care"[Mesh:NoExp] OR "integrated care"[tiab] OR "comprehensive care*"[tiab] OR "collaborative care"[tiab] OR "Patient Centered Care"[tiab] OR "Person Centered Care"[tiab] OR "Patient-Focused Care"[tiab] OR "Patient Centered Nursing"[tiab] OR "Medical Home*"[tiab] OR "Progressive Patient Care*"[tiab] OR "Continuity of Patient Care"[tiab] OR "Continuum of Care"[tiab] OR "Care Continuum"[tiab] OR "Continuity of Care"[tiab] OR "Care Continuity"[tiab] OR "Case Management"[Mesh] OR "Patient Care Planning"[Mesh:NoExp] OR "Patient Care Plan*"[tiab] OR "Nursing Care Plan*"[tiab] OR "Goals of Care"[tiab] OR "Care Goal*"[tiab] OR "comprehensive healthcare"[tiab] OR "comprehensive health care"[tiab] OR "integrated healthcare"[tiab] OR "integrated health care"[tiab] OR "collaborative healthcare"[tiab] OR "collaborative health care"[tiab] OR "comprehensive model"[tiab] OR "collaborative model"[tiab] OR "integrated model"[tiab] OR “integrated delivery of health”[tiab] OR “collaborative patient care”[tiab] OR “integrated patient care”[tiab] OR “comprehensive patient care”[tiab]

6 Annex II: Study Characteristics

Care model	Study	Country	Design	Inclusion criteria	Duration of intervention	Setting	Cohort	N
BRIGHTEN	Emery 2012	USA	Program evaluation	65+ in first 3 years; funder required change to 60+ in Year 4 of the program	Follow-up at 6 months	Outpatient clinics		150
	Emery 2011	USA	Program evaluation	BRIGHTEN team members: gerontopsychologist, geriatric social worker, geriatric psychiatrist, occupational therapist, physical therapist, nutritionist, and chaplain				
CAREPATH	Bruce 2011, 2015	USA	Cluster randomized effectiveness trial	≥65 years Medicare Home Health patients who screen positive for depression	Follow-up at 3,6 and 12 months	Home health care agencies		306
CASPER plus	Overend 2014, Bosanquet 2017	UK	RCT	≥65 years screen-positive for depression on the recommended QOF 2 question brief depression screen	8 weeks, follow-up at 4, 12 and 18 months	Primary care [GP practices]		485
	Taylor 2018	UK	Qualitative interviews	GPs within CASPER plus trial practices, case managers delivering the intervention, and patients [including both participants who completed, and those who withdrew from the intervention]				GP = 12 CM = 8 P = 11
CEPIS	Ng 2020	Singapore	RCT	≥60 years Depressive symptoms according to 15-items GDS	6 months, follow-up at 3,6 and 12 months	Community-based		214
	Nyunt 2009	Singapore	Observational study	>60 years Regular use of social services				4633
DCM project	Chen 2011, 2015	China	Cluster-randomised trial	≥60 years Major depression Living independently	16 weeks, follow-up at 3,6 and 12 months	Primary center clinics		326
GermanIMPACT	Wernher 2014	Germany	Study protocol	≥60 years Moderate depressive symptoms in the scope of a diagnosed depressive episode, recurrent depressive disorder, or dysthymia [ICD-10]	12 months, follow-up at 6 and 12 months	Primary care [GP practices]		300

				PHQ-9; score 10 to 14				
	Kloppe 2020	Germany	Explanatory sequential design	≥60 years<br>Major depression, dysthymic disorder or both			12 month intervention patients	26
IMPACT	Fann 2009	USA	RCT	≥60 years<br>Current major depression or dysthymia [chronic depression]<br>Plans to use one of the participating clinics as the main source of general medical services for the coming year	12 months, follow-up at 3, 6, 12, 18 and 24 months	Primary care clinics	Patients with cancer	215
	Harpole 2005	USA	Preplanned analyses of a RCT	≥60 years<br>Current major depression or dysthymia<br>Plans to use one participating primary care clinic as the main source of general medical services for the coming year	12 months, follow-up at 3, 6 and 12 months	Primary care clinics	July 1999 to August 2001	1801
	Hunkeler 2006	USA	RCT	≥60 years<br>Major depression or dysthymia, or both, according to SCID	12 months, follow-up at 18 and 24 months	Primary care clinics	July 1999 to August 2001	1801
	Khambaty 2018	USA	Post-trial follow-up RCT	>60 years<br>Current major depressive disorder or dysthymia diagnosis	12 months, 9-year follow-up	Primary care clinics	Patients from Indiana with unique set of resources–i.e., local electronic medical record data [including death certificate data] linked with Medicare and Medicaid claims	119
	Lin 2006	USA	RCT	>60 years<br>Met criteria for major depression and/or dysthymia	12 months, follow-up at 12 months	Primary care clinics	Patients with arthritis	105
	Lin 2003	USA	RCT	≥60 years<br>Current major depression or dysthymia diagnosed by the DSM-IV<br>Plans to use one participating primary care clinic as the main source	12 months, follow-up at 3, 6 and 12 months	Primary care clinics	Patients with arthritis	1001

				of general medical services for the coming year				
	Callahan 2005	USA	Multisite randomized clinical trial	≥60 years<br>Intention to use one of the participating clinics as the main source of general medical care in the coming year<br>Diagnosis of current major depression or dysthymic disorder	12 months, follow-up at 3, 6 and 12 months	Primary care clinics	July 1999 to August 2001	1801
	Van Leeuwen-Williams 2009	USA	RCT	≥60 years<br>Intent to use one of the study clinics as the main source of medical care during the next year<br>Diagnosis of current major depression or dysthymia	12 months, follow-up at 3, 6, 8, 12, 18 and 24 months	Primary care clinics	IV patients	906
	Oishi 2003	USA	Qualitative [focus groups & interviews]	Depression Clinical Specialists [DCSs] working with Project IMPACT		Primary care clinics	DCSs	11
	Penkunas 2015	USA	Retrospective evaluation study	≥60 years<br>with substantial symptoms of depression, were at or below 300 % of the Federal Poverty Level, insured through MediCal, dually covered under MediCal and MediCare, or uninsured		Primary care clinics	August 2009 and February 2013	112
	Riebe 2012	USA	Observational mixed methods analysis	≥60 years<br>Intent to use one of the study clinics as the main source of medical care during the next year<br>Diagnosis of current major depression or dysthymia		Primary care clinics	IV patients with documented session notes	597
	Williams 2004	USA	Preplanned subgroup analysis of RCT	≥60 years<br>Plans to use one of the participating clinics as the main source of general medical care in the coming year<br>Diagnosis of current major depression or dysthymic disorder	12 months, follow-up at 3, 6 and 12 months	Primary care clinics	Co-existing diabetes	417
	Thielke 2007	USA	Secondary data analysis	≥60 years<br>Diagnosis of current major depression or dysthymia according to DSM-V		Primary care clinics	July 1999 to August 2001	1801
	Unutzer 2002	USA	RCT	≥60 years<br>Major depression,	12 months, follow-up at	Primary care clinics	July 1999 to August 2001	1801

				dysthymic disorder or both	3, 6 and 12 months			
IMPACT-DP	Unutzer 2008	USA	Pilot study	≥60 years<br>ICD-9 diagnosis of osteoarthritis in the prior year<br>Two-item screen for depression	6 months, follow-up at 6 months	Primary care clinics		13
Improving Depression Treatment for Older Minority Adults in Public Sector Care	Camacho 2018	USA	Qualitative interviews	Older Hispanic immigrants<br>GDS-15 score ≥5<br>PHQ-9; ≥10		Specialty geriatric clinic, providing primary services		14
Multifaceted shared care intervention for late life depression in residential care	Llewelyn Jones 1999	Australia	RCT	>65 years<br>Cognitively able to provide accurate information were screened for depression	Follow-up at 9.5 months	Residential facility		220
PRIDE	Chew-Graham 2007	UK	RCT	>60 years<br>Clinically identified as depressed	12 weeks, follow-up at 16 weeks	Primary care trust		105
PROSPECT	Alexopoulos 2005	USA	RCT	≥60 years<br>CES–D score >20<br>a 5% random sample of patients with lower scores	Follow-up at 4, 8, 12 and 18 months	Primary care practices	Patients who have been evaluated at 4 month follow-up visit	215
	Alexopoulos 2009	USA	RCT	≥60 years<br>Major depression [DSM-IV] OR<br>Minor depression: 3-4 depressive symptoms, ≥ 10 on the 24-item HAM-D, and a duration of at least 1 month	Follow-up at 4, 8, 12, 18 and 24 months	Primary care practices	May 1999 through August 2001	599
	Bogner 2005	USA	Multisite effectiveness trial	≥60 years<br>CES–D score >20<br>a 5% random sample of patients with lower scores	Follow-up at 4,8 and 12 months	Primary care practices	MDD	324
	Bogner 2007	USA	RCT	60–74 years or ≥ 75 years<br>CES-D score >20<br>a 5% random sample of patients with lower scores OR<br>CES-D score ≤20 + supplemental questions about mood, prior depressive episodes, or treatment	Follow-up at 70 months	Primary care practices	May 1999 through August 2001	584
	Bogner 2012	USA	RCT	≥60 years<br>Community dwelling<br>Attending a scheduled visit with a PCP<br>CES-D >20<br>a 5% random sample of patients with CES-D < 20	Follow-up at 4, 8, 12 and 24 months	Primary care practices	May 1999 through August 2001	599

	Bruce 2004	USA	RCT	≥60 years<br>MMSE score ≥18<br>CES-D >20<br>a 5% random sample of patients with CES-D < 20	Follow-up at 4, 8 and 12 months	Primary care practices	May 1999 through August 2001	598
	Schulberg 2007	USA	Secondary analysis PROSPECT [RCT]	>60 years<br>Regularly scheduled primary care appointments<br>CES-D >20<br>a 5% random sample of those scoring below this CES-D threshold			12 month IV patients	320
The Seniors Outpatient Community-Based Collaborative Care Model	Shulman 2021	Canada	Feasibility study	≥65 years<br>Anxiety and or depression symptoms<br>≥1 chronic physical health condition impacting function	3 months post discharge follow-up	Community-based		187

Abbreviations Annex I

BRIGHTEN: Bridging Resources of an Interdisciplinary Geriatric Health Team via Electronic Networking
CAREPATH: [Depression] CARE for PATients at Home
CASPER plus: CollAborative care in Screen-PositiveEldeRs with major depressive disorder
CEPIS: Community-Based Early Psychiatric Interventional Strategy
CES-D: Center for Epidemiologic Studies Depression scale
DCM project: Depression Care Management
DSM: Diagnostic and Statistical Manual of Mental Disorders
GDS: Geriatric Depression Scale
GP: General Practitioner
HAM-D: Hamilton Rating Scale for Depression
ICD: International Statistical Classification of Diseases and Related Health Problems
IMPACT: Improving Mood Promoting Access to Collaborative Care Treatment
MMSE: Mini-Mental State Examinition
PCP: Primary Care Provider/Physician
PHQ-9: Patient Health Questionnaire
PRIDE: Primary care Intervention for Depression in the Elderly
PROSPECT: Prevention of Suicide in Primary Care El-derly: Collaborative Trial
QOF: Quality and Outcomes Framework
SCID: Structured Clinical Interview for DSM Disorders
